# Chicken adaptive response to low energy diet: main role of the hypothalamic lipid metabolism revealed by a phenotypic and multi-tissue transcriptomic approach

**DOI:** 10.1186/s12864-019-6384-8

**Published:** 2019-12-30

**Authors:** F. Jehl, C. Désert, C. Klopp, M. Brenet, A. Rau, S. Leroux, M. Boutin, L. Lagoutte, K. Muret, Y. Blum, D. Esquerré, D. Gourichon, T. Burlot, A. Collin, F. Pitel, A. Benani, T. Zerjal, S. Lagarrigue

**Affiliations:** 1 PEGASE UMR 1348, INRA, AGROCAMPUS OUEST, 35590 Saint-Gilles, France; 2 SIGENAE Plateform, INRA, 31326 Castanet-Tolosan, France; 3 GABI UMR 1313, INRA, AgroParisTech, Université Paris-Saclay, 78350 Jouy-en-Josas, France; 4 GenPhySE UMR 1388, INRA, INPT, ENVT, Université de Toulouse, 31326 Castanet-Tolosan, France; 5 Programme Cartes d’Identité des Tumeurs (CIT), Ligue Nationale Contre Le Cancer, 75013 Paris, France; 6 GENOTOUL Plateform, INRA, 31326 Castanet-Tolosan, France; 7 PEAT UE, INRA, 37380 Nouzilly, France; 8 NOVOGEN, Mauguérand, 22800 Le Foeil, France; 9 BOA UMR, INRA, Université de Tours, 37380 Nouzilly, France; 10 Centre des Sciences du Goût et de l’Alimentation, AgroSup Dijon, CNRS, INRA, Université de Bourgogne, Dijon, France

**Keywords:** Transcriptome, Lipid, Feed intake, Adaptation, Hypothalamus, Chicken

## Abstract

**Background:**

Production conditions of layer chicken can vary in terms of temperature or diet energy content compared to the controlled environment where pure-bred selection is undertaken. The aim of this study was to better understand the long-term effects of a 15%-energy depleted diet on egg-production, energy homeostasis and metabolism via a multi-tissue transcriptomic analysis. Study was designed to compare effects of the nutritional intervention in two layer chicken lines divergently selected for residual feed intake.

**Results:**

Chicken adapted to the diet in terms of production by significantly increasing their feed intake and decreasing their body weight and body fat composition, while their egg production was unchanged. No significant interaction was observed between diet and line for the production traits. The low energy diet had no effect on adipose tissue and liver transcriptomes. By contrast, the nutritional challenge affected the blood transcriptome and, more severely, the hypothalamus transcriptome which displayed 2700 differentially expressed genes. In this tissue, the low-energy diet lead to an over-expression of genes related to endocannabinoid signaling (*CN1R*, *NAPE-PLD*) and to the complement system, a part of the immune system, both known to regulate feed intake. Both mechanisms are associated to genes related polyunsaturated fatty acids synthesis (*FADS1*, *ELOVL5* and *FADS2*), like the arachidonic acid, a precursor of anandamide, a key endocannabinoid, and of prostaglandins, that mediate the regulatory effects of the complement system. A possible regulatory role of *NR1H3* (alias *LXRα*) has been associated to these transcriptional changes. The low-energy diet further affected brain plasticity-related genes involved in the cholesterol synthesis and in the synaptic activity, revealing a link between nutrition and brain plasticity. It upregulated genes related to protein synthesis, mitochondrial oxidative phosphorylation and fatty acid oxidation in the hypothalamus, suggesting reorganization in nutrient utilization and biological synthesis in this brain area.

**Conclusions:**

We observed a complex transcriptome modulation in the hypothalamus of chicken in response to low-energy diet suggesting numerous changes in synaptic plasticity, endocannabinoid regulation, neurotransmission, lipid metabolism, mitochondrial activity and protein synthesis. This global transcriptomic reprogramming could explain the adaptive behavioral response (i.e. increase of feed intake) of the animals to the low-energy content of the diet.

## Background

The egg-production sector uses genetically selected chicken breeds bought from a few breeding companies. While the purebred selection process usually takes place in a controlled environment, commercial layers are exposed to a wide diversity of environments, some being more challenging than others because of stressors like high heat, sub-optimal diet composition or low diet energy content. In this study we investigated, on laying hens, the effects that a 15%-energy depleted diet provided ad libitum over a long period (14 weeks) has on the transcriptome of several energy-related tissues to verify if animal performance changes related to the low energy intake were due to underlying mechanisms at the transcriptomic level. The low-energy diet used in this study resembles the type of diet that can used for layer production in countries where, for diverse reasons, access to protein or oil happens to be too costly or impossible due to the lack of supply. While several studies have investigated the effect of a low-energy diet on the performances of laying hens, no study has analyzed the tissue mechanisms underlying performance variations at the transcriptomic level. As examples, Grobas et al. [1] observed an increase in feed intake, a decrease in body weight gain and no difference in egg production rate and egg weight in layers fed ad libitum a 2680 kcal/kg diet compared to a 2810 kcal/kg diet, both with the same protein content levels per kilocalorie of energy, from 22 to 65 weeks of age. Harms et al. [2] observed the same results regarding feed intake, body weight gain, egg production rate and egg weight for layers fed a 2519 kcal/kg diet compared to a 2798 kcal/kg control diet from 36 to 44 weeks of age, with adjusted levels of amino-acids. On the contrary, Murugesan and Persia [3] observed no effects on egg production, body weight and feed intake, but only a reduction of the abdominal fat pad mass in layers fed ad libitum a 2790 kcal/kg diet, compared to a 2880 kcal/kg control diet, both diets having approximately the same crude proteins content, from 28 to 39 weeks In this context, we investigated the effects of a low-energy diet on the performances and feed intake together with the transcriptomes of four tissue of adult layers fed ad libitum two diets differing in energy content (2321 kcal/kg for the low-energy diet versus 2710 kcal/kg for the commercial diet) from 17 to 31 weeks of age. Since feed efficiency is a key factor for energy allocation and is a trait of economic importance, we hypothesized a possible interaction between feed efficiency and the response to the energy-depleted diet. We therefore compared the response to the low-energy diet between two brown egg layer lines divergently selected for the residual feed intake (RFI) [4] to evaluate such a potential interaction between diet and feed efficiency factors. The RFI is the difference between the predicted feed intake estimated considering body weight and egg production, and the observed feed intake. The four tissues used to explore the transcriptomic mechanisms at work in response to the low-energy diet on the same animals as those used for the performance analysis were the liver, the adipose tissue, the blood and the hypothalamus, all related to energy homeostasis. The adipose tissue is crucial for fatty acid storage, the main form of energy storage, and mobilization. The liver is a key organ for lipogenesis in birds [5], in addition to many other physiological processes such as oxidation, secretion and detoxification. The hypothalamus is an important center for the regulation of feed intake, and blood is a circulating tissue that gathers and transports nutrients, hormones, proteins and cell waste throughout an organism. To the best of our knowledge, such a study analyzing both laying performances and four tissue transcriptomes in response to an energy-depleted diet has not yet been undertaken in layers.

## Results

### Diet energy change had little effect on production traits but affected feed intake and body composition

The line, diet and interaction effects on body weight, egg production and shell strength, feed intake (FI), residual feed intake (RFI) and abdominal adipose weight after 14 weeks of the low-energy diet are summarized in Table 1. The diet energy content difference had no effect on egg production, i.e. on laying rate, egg weight and egg mass. In contrast, we observed a significant decrease in body weight at 31 weeks (on average for both lines, − 4.4%, *p* < 0.05) in the LE group compared to the CT group, despite the fact that at the beginning of the trial (17 weeks of age), the LE group was slightly heavier than the CT group (on average, + 3%, *p* < 0.05, Additional file 1). We also observed a significant (*p* < 0.05) increase of feed intake in the LE group over 28 to 31th week of age, without significant interaction with the line (*p* = 0.50). It can however be noted that the increase in feed intake in response to the LE diet is smaller in the R- line (+ 145 g) than in the R+ line (+ 270 g), which can be related to the fact that the R- line generally eats less; the interaction between diet and line remains however not significant. The calculated RFI was significantly higher in the LE group, meaning that the animals were less feed efficient than the CT group. Finally, the LE group had at 31 weeks of age a significantly lower ratio of abdominal adipose tissue weight to body weight compared to the CT group (on average, − 0.72, *p* < 0.05), even if the body weight significantly decreased at the same age (on average − 4.4%, *p* < 0.05) indicating a higher decrease of abdominal tissue (on average, − 20.6%, *p* < 0.05). Concerning the line factor, as expected, we observed significant differences on FI, RFI and abdominal adipose weight. The significant line effect for the body weight at 31 weeks, for which the interaction *p*-value was the lowest and close to 0.10 is due to the {R-,LE} group, the animals of which are lighter than in the three other groups. However, we observed no significant differences between the body weight of R+ and R- from the control group, as expected since the divergent selection on RFI was performed at constant body weight. Both lines, regardless of their RFI, reacted in a similar way to the energy-depleted diet by increasing their feed intake. However, this increase in feed ingestion was not sufficient to avoid body weight loss in the R- fed with the LE diet and depletion of the energy reserves (body fat). To explore the molecular mechanisms underlying this adaptation, we analyzed gene expression of several tissues of birds from these two lines and diets.

**Table 1:** Means (±SD) and significance for production, feed efficiency and body composition traits, for the effect of the diet, the line and their interaction

	{R+,CT}^a^	{R+,LE}^a^	{R-,CT}^a^	{R-,LE}^a^	Diet^b^	Line^b^	Diet × Line^b^
Body weight, week 31 (g)	2162.35 (±165.33)	2142.46 (±129.28)	2089.44 (±216.87)	1925.40 (±217.32)	*	**	0.11
Laying intensity (%)	86.17 (±11.92)	87.73 (±7.81)	86.87 (±5.44)	84.59 (±8.58)	0.70	0.50	0.54
Egg number	60.94 (±9.33)	62.18 (±9.93)	61.17 (±6.16)	60.47 (±7.43)	0.93	0.86	0.60
Egg weight (g)	47.91 (±3.11)	46.80 (±2.98)	48.08 (±2.25)	47.61 (±1.82)	0.21	0.53	0.60
Egg mass (g)^c^	1166.41 (±181.31)	1182.36 (±210.53)	1118.36 (±108.85)	1055.80 (±126.99)	0.43	*	0.27
Static stiffness (N.mm^−1^)	109.68 (±18.75)	104.64 (±15.58)	126.75 (±18.39)	118.95 (±18.76)	0.12	***	0.75
Feed intake (g)^c^	4128.47 (±426.94)	4398.10 (±551.14)	2583.92 (±308.26)	2728.73 (±419.65)	*	***	0.50
Energy intake (kcal)^c^	11,188.16 (±1157.00)	10,207.97 (±1279.19)	7002.41 (±835.38)	6333.39 (±974.01)	**	***	0.52
RFI (g/21d^−1^)^c^	868.36 (±329.66)	1152.32 (±390.52)	− 614.35 (±134.93)	− 196.81 (±211.78)	***	***	0.28
Abdominal adipose weight at 31 weeks (g)	73.33 (±21.10)	57.10 (±18.61)	129.83 (±44.23)	105.00 (±31.67)	*	***	0.64
Ratio of abdominal adipose weight to body weight at 31 weeks (%)	3.37 (±0.83)	2.65 (±0.78)	5.96 (±1.39)	5.24 (±1.09)	*	***	1

^a^Values represent the line/treatment group means for each trait (±standard deviation). R+ refers to low feed efficient layers and R- to high feed efficient layers, CT to control group and LE to low energy diet. The number of animals analyzed are: R+,CT *n* = 34, R+,LE *n* = 11, R-,CT *n* = 36, R-,LE *n* = 15

^b^***: *p* < 0.001, **: *p* < 0.01, *: *p* < 0.05

^c^Feed-related traits were measured between 28 and 31 weeks of age

### Diet energy change leads to transcriptomic modifications, mainly in hypothalamus and blood

**Fig. 1:**
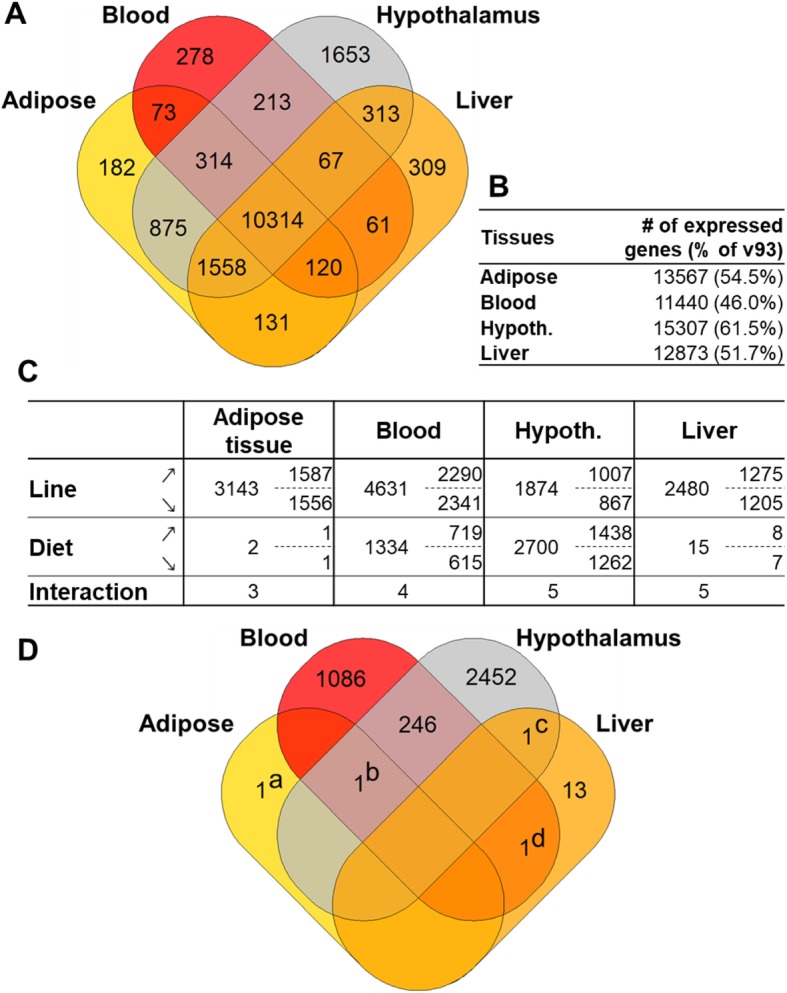
Overview of gene expression and differential expression between diets in the adipose tissue, blood, hypothalamus and liver. **a** Venn diagram of the genes expressed and shared in the four tissues. **b** Total number of genes expressed in each tissue; between brackets, percentage of v87 annotation (24,881 genes). **c** Differentially expressed genes (DEG) in each tissue (columns) and each factors, Line, Diet and Interaction (rows). The total number of DEG (left) and the details of the number of up- (↗) and down-expressed genes (↘) in LE diet (or R+ line) compared to CT (to R- line) are indicated. Hypoth.: Hypothalamus. **d** Venn diagram of the DEG between diets in the four tissues. Single genes in the diagram are: (a) ENSGALG00000002503 (SFTPA2) (b) ENSGALG00000031497 (no HGNC), (c) ENSGALG00000026507 (FDX1) and (d) ENSGALG00000006099 (ZFPM1)

To explore the genes involved in the response of birds to the two diets, we analyzed the transcriptomic changes associated with diet changes in the adipose tissue, blood, hypothalamus and liver. A total of 16,461 genes were expressed in at least one of the four tissues considered, and represents 66% of the 24,881 genes from Ensembl v93 annotation (Fig. 1a and b). Of these 16,461 genes, 13,567, 11,440, 15,307 and 12,873 were expressed in the adipose, blood, hypothalamus and liver, respectively (Fig. 1b), and 10,314 (41%) were expressed in all four tissues (Fig. 1a). Some of these genes were tissue-specific, representing 1.34% (adipose) to 10.8% (hypothalamus) of the total number of genes expressed in the tissues (Fig. 1a, Additional file 2). The hypothalamus had markedly higher gene-specificity, with 1653 genes expressed only in this tissue. It also had the greatest number of total expressed genes (15307). Strikingly, diet change had a large effect on the hypothalamic and blood transcriptomes, with 2700 and 1334 differentially expressed genes (DEG), respectively, while the hepatic and adipose tissue transcriptomes were almost unaffected (15 and 2 DEG, respectively) (Fig. 1c and d, Additional file 3). The line had a major effect in all tissues, with 3143, 4631, 1874 and 2480 DEG in the adipose, blood, hypothalamus and liver, respectively. As only a very small number of significant interactions (*p*_*FDR*_ < 0.05) were observed (Fig. 1c), allowing for an independent analysis of the line and diet factors, the present paper focuses only on the diet effect.

### Functional characterization of hypothalamic transcriptome changes upon diet energy challenge

Among the 2700 DEG detected in the hypothalamus in response to the diet energy change, 1438 and 1262 genes were over- and under-expressed, respectively, in the LE group compared to the control. We characterized these two DEG lists using KEGG pathway term enrichment as described in Methods. For the over- and under-expressed gene lists, 26 and 44 pathways (*p*_*FDR*_ < 0.05) were significantly enriched (Additional file 4). The 10 top terms with the lowest *p*_*FDR*_ for both DEG lists are presented in Table 2.

**Table 2:** Top 10 (based on *p*_*FDR*_) KEGG pathways associated with under-expressed (**A**) and over-expressed DEG (B) in the hypothalamus

Term	# of genes	*p*_*FDR*_
A. Under-expressed genes in LE compared to CT		
Synaptic vesicle cycle	22	7.36 × 10^−11^
Glutamatergic synapse	26	1.79 × 10^−08^
Dopaminergic synapse	26	2.37 × 10^−07^
Axon guidance	25	5.62 × 10^−07^
Oxytocin signaling pathway	27	2.46 × 10^−06^
Circadian entrainment	20	2.50 × 10^−06^
Oocyte meiosis	21	7.03 × 10^− 06^
Protein processing in endoplasmic reticulum	26	2.04 × 10^−05^
Nicotine addiction	12	2.04 × 10^−05^
GABAergic synapse	17	5.18 × 10^− 05^
B. Over-expressed genes in LE compared to CT		
Ribosome	83	1.03 × 10^−67^
Metabolic pathways	166	2.57 × 10^−25^
Oxidative phosphorylation	46	3.26 × 10^−22^
Glycine, serine and threonine metabolism	15	7.73 × 10^−08^
Fatty acid metabolism	15	1.81 × 10^−06^
Fatty acid degradation	14	2.52 × 10^−06^
Valine, leucine and isoleucine degradation	14	3.18 × 10^−06^
PPAR signaling pathway	16	3.65 × 10^−05^
Carbon metabolism	19	1.54 × 10^−04^
Alanine, aspartate and glutamate metabolism	10	4.70 × 10^−04^

Pathways associated with the under-expressed genes (Table 2A) comprised 91 under-expressed genes related to different types of synapses: glutamatergic, dopaminergic and GABAergic synapses, as well as the synaptic vesicle cycle or axon guidance. Among these genes were notably *GRIA1*, *GRIA3* and *GRIA4* that code for subunits of the glutamate receptor, the predominant excitatory neurotransmitter in the nervous system; *DDC*, that code for an enzyme involved in the synthesis of dopamine, a neurotransmitter involved in the reward system, and *DRD3* that code for a subunit of the dopamine receptor; *GABRQ*, *GABRG2*, *GABRR2* that code for subunits of the receptor to the gamma-aminobutyric acid (GABA), the major inhibitory neurotransmitter.

**Fig. 2:**
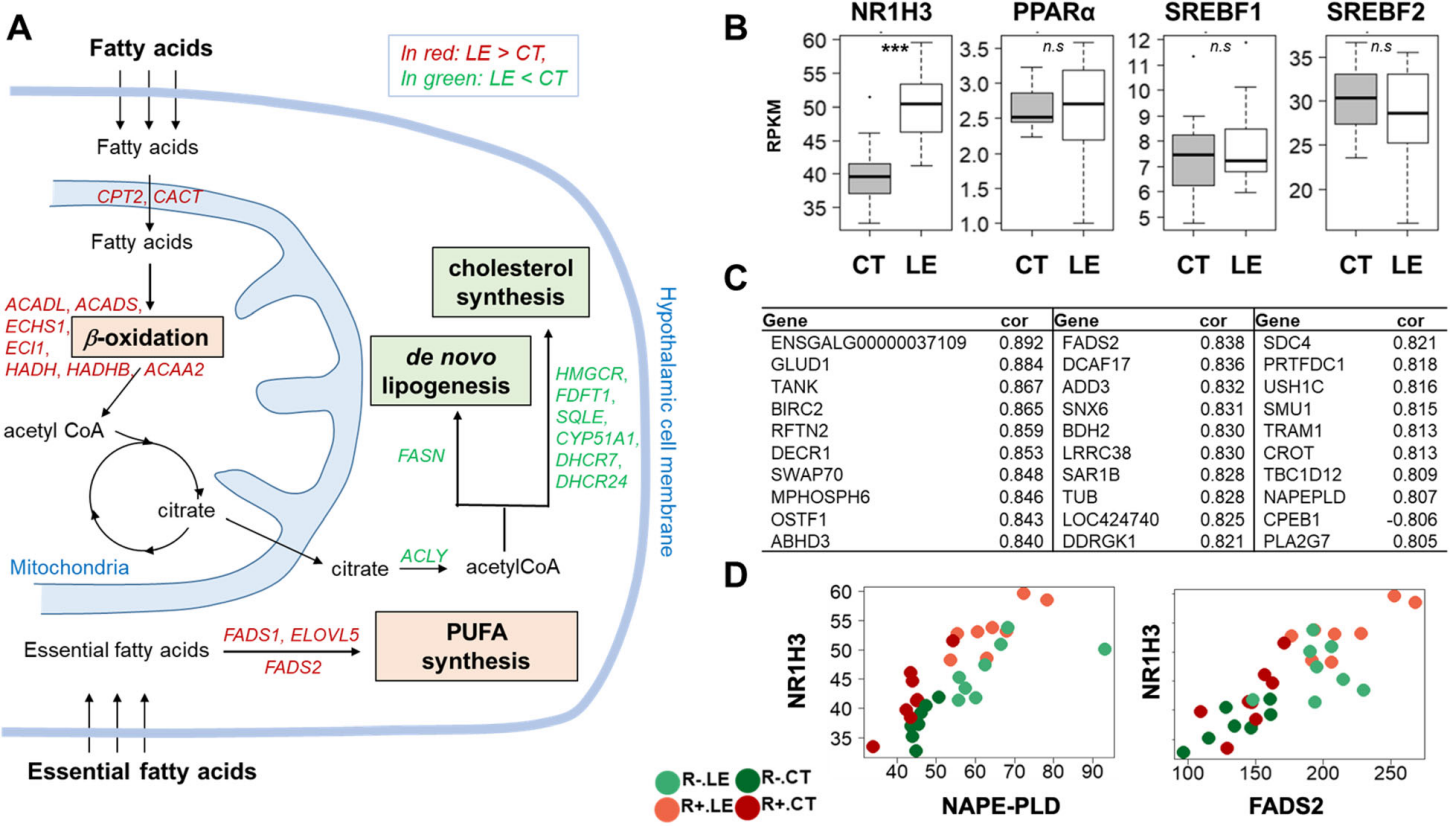
Lipid metabolism modulation in the hypothalamus in response to the LE diet and genes highly correlated to NR1H3 (LXRα). **a** Schematic summary of the lipid metabolism related genes found to be differentially expressed in the hypothalamus of LE group. **b** Boxplot of the expression of the key lipid transcription factor/nuclear receptors. **c** Top 30 genes which expression is correlated to NR1H3. **d** Co-expression plot of NR1H3 with NAPE-PLD (right) and FADS2 (left). *n.s*: not significant; ***: *p* < 0.001

Pathways associated with over-expressed genes in LE compared to CT (Table 2B) were related to the “Ribosome” and several metabolic pathways. “Ribosome” comprises 83 ribosomal Protein genes, of which 41 Ribosomal Protein L (*RPLx*) genes, 27 Ribosomal Protein S (*RPSx*), as well as 8 Mitochondrial Ribosomal Protein L (*MRPLx*) and 5 Mitochondrial Ribosomal Protein S (*MRPSx*). Among the metabolic pathways, energy-related pathways appear to be most affected. Indeed, we found an over-representation of genes associated with oxidative phosphorylation, a process that involves a series of oxidation-reduction reactions in mitochondria, resulting in the phosphorylation of ADP to produce ATP. Among these genes, 31 were related to one of the 5 protein complexes constituting the respiratory chain located in the inner mitochondrial membrane: 15 genes for the complex I (NADH:ubiquinone oxidoreductase), 8 genes the complex II (succinate:ubiquinone oxidoreductase), 3 genes for the complex III (ubiquinol:ferricytochrome C oxidoreductase), 2 genes for the complex IV (cytochrome C oxidase) and 2 genes for the complex V (FoF1-ATP synthetase), in addition to SLC25A4, the ADP/ATP translocase 1. More than 21 of them are located in the mitochondrial genome. In addition, genes involved in fatty acid transport (*ACSBG1*, *APOA1*, *APOC3*, *DBI*, *SLC27A1*, *FABP4*, *FABP7*, *SCP2*), the fatty acid β-oxidation in the mitochondria (*CPT2*, *CACT*, *ACADL*, *ACADS*, *ECHS1*, *ECI1*, *HADH*, *HADHB*, *ACAA2*), and to a lesser extent, in the peroxisomes (*ACAA1*, *ACOX*, *ECI2*) were also over-expressed. On the contrary, genes involved in the de novo lipogenesis were significantly under-expressed, in particular *FASN*, that codes for a key enzyme of the saturated fatty acid synthesis, and *ACLY* that codes for the primary enzyme involved in the synthesis of cytosolic acetyl-CoA from citrate. Similarly genes involved in the cholesterol synthesis such as *HMGCR*, *FDFT1*, *SQLE, CYP51A1, DHCR7*, and *DHCR24* were also under-expressed*.* Interestingly, we observed an over-expression of genes involved in the biosynthesis of ω3 and ω6 polyunsaturated fatty acids, with *FADS2*, *ELOVL5, FADS1*, *ELOVL2* and (see top 5 and 19 KEGG term). It is noteworthy that one of the products of this pathway, the arachidonic acid, can be used by the enzyme coded by *NAPEPLD*, which is over-expressed (FC = 1.93, *pFDR* = 6.86 × 10^− 11^) as a substrate for the synthesis of anandamide. Since the lipid metabolism was largely impacted (Fig. 2a), we studied the transcription factors related to this metabolism (Fig. 2b). The expressions of *PPARA*, *SREBF2* and *SREBF1* genes were not affected (FC = 1; 0.88 and 1.08 respectively, with *p*_*FDR*_ = 0.99; 0.44 and 0.79, respectively). On the other hand, *NR1H3* (alias *LXRA*) was significantly over-expressed (FC = 1.55, *p*_*FDR*_ = 2 × 10^− 6^). The 30 genes most correlated (*r* > 0.8) to *NR1H3* are showed in Fig. 2c in which can be found *FADS2* and *NAPE-PLD* (*r* = 0.81 and *r* = 0.84, *p*_*FDR*_ < 2.24 × 10^− 5^ and *p*_*FDR*_ < 5.4 × 10^− 6^, respectively, Fig. 2d).

### Functional characterization of blood transcriptomic changes upon diet energy change

Among the 1334 DEG detected in the blood in response to the dietary change, 719 and 615 genes were over- and under-expressed, respectively, in the LE compared to the CT group. KEGG characterization of the over- and under-expressed DEG lists reveals 2 and 8 significantly enriched pathways, respectively (*p*_*FDR*_ < 0.05) (Additional file 5). The terms for both DEG lists are presented in Table 3.

**Table 3:** KEGG pathways associated with over-expressed (A) and under-expressed DEG (B) in the blood

Term	# of genes	*p*_*FDR*_
A. Under-expressed genes in LE compared to CT		
Metabolic pathways	61	7.92 × 10^−05^
Biosynthesis of amino acids	10	2.18 × 10^−03^
Carbon metabolism	11	8.02 × 10^−03^
Fructose and mannose metabolism	6	9.32 × 10^−03^
Steroid biosynthesis	5	9.32 × 10^− 03^
Amino sugar and nucleotide sugar metabolism	7	9.32 × 10^−03^
Pentose phosphate pathway	5	2.20 × 10^−02^
Galactose metabolism	5	3.82 × 10^−02^
B. Over-expressed genes in LE compared to CT		
Ribosome	13	2.95 × 10^−02^
RNA degradation	9	3.24 × 10^− 02^

The pathways associated with under-expressed genes in blood are related to “Metabolic pathways”, in particular amino acids biosynthesis (*ACO2*, *ALDH7A1*, *CPS1*, *CTH*, *ENO2*, *GOT1*, *PFKP*, *TALDO1*, *TKT*, *TPI1*), fructose and mannose metabolism (*AKR1B1*, *AKR1B10*, *PFKFB4*, *PFKP*, *PMM2*, *TPI1*) or galactose metabolism (*AKR1B1*, *AKR1B10*, *GALK2*, *PFKP*, *PGM2*). Genes involved in cholesterol biosynthesis were under-expressed in blood (*FDFT1*, *SQLE*, *CYP51A1*, *NSDHL* and *DHCR24*) as in hypothalamus. The two pathways associated with over-expressed genes are “RNA degradation”, with *EDC3*, *EXOSC5*, *PABPC1*, *PAN2*, *PAN3*, *PATL1*, *RQCD1*, *SKIV2L* and *TOB2*, and “Ribosome”, which contains 3 RPL, 3 MRPL, 3 Ribosomal Protein Lateral Stalk Subunit P (RPLP*x*) and 4 RPS genes, 11 out of these 13 genes were also over-expressed in hypothalamus.

### Detection of co-expressed genes with WGCNA within hypothalamus and blood DEG lists

**Fig. 3:**
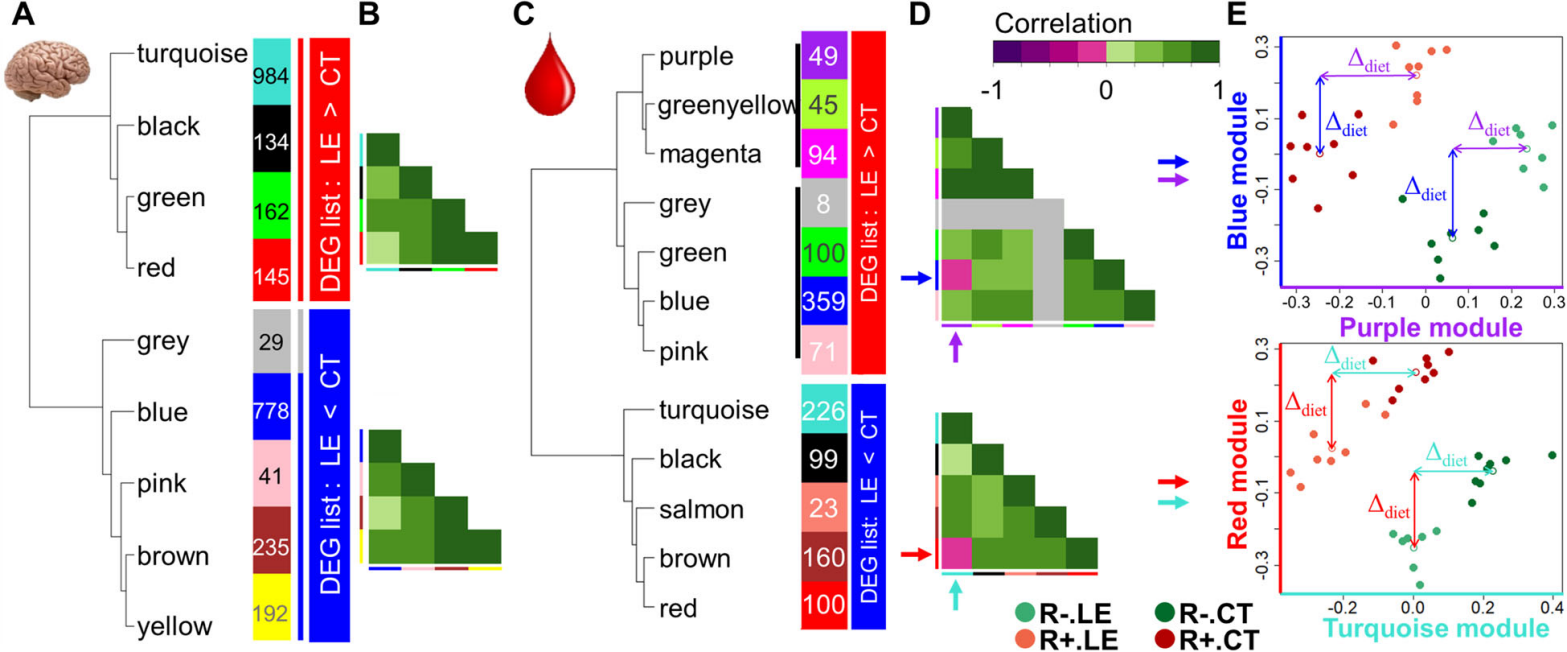
Analysis of WGCNA modules obtained for the hypothalamus and blood differentially expressed genes. Hierarchical clustering of the eigengenes of the modules detected with hypothalamus (**a**) and blood (**b**) DEG. Module colors are drawn next to module names, with the number of genes in the modules. Unclustered genes are in the grey module. The boxes on the right indicate whether the module contains over-expressed (LE > CT) genes (red) or under-expressed (LE < CT) genes (blue). Black lines highlight the 2 subsets distinguished by WGCNA for the LE > CT DEG list. **c** Heatmap of the correlation matrix between the modules eigengenes. Note the negative correlation (pink boxes) between the purple and blue modules (*top*) and turquoise and red modules (*bottom*). **d** Plots of two pairs of module eigengenes from blood DEG. *Top*: purple vs. blue module from the LE < CT DEG list, *bottom*: turquoise vs. red module from the LE > CT DEG list. Δ_diet_ is the difference between the LE mean vs. CT mean (symbolized with an empty circle) for each line

To detect gene subsets in our DEG lists, we used the R package WGCNA to identify and cluster co-expressed gene modules (see Methods). As shown in Fig. 3, WGCNA separated for hypothalamus (Fig. 3a) and blood (Fig. 3c) different co-expression groups (noted by a color) for both “LE > CT” (in red) and “LE < CT” (in blue) DEG lists. Interestingly, 2 modules of the same DEG list were not positively correlated in the blood (Fig. 3d, pink color in the correlation matrix) with the blue and purple modules for the red “LE > CT” DEG list and the red and turquoise modules of the blue “LE < CT” DEG list, while all modules were positively correlated in the hypothalamus (Fig. 3b). The plots of module eigengenes of these two pairs can be found in Fig. 3e. We can clearly distinguish in the two plots, two distinct parallel series of points that correspond to the R+ and R- lines. This parallelism reveals two facts: first, a difference of expression between the lines with a positive “R- / R+” expression ratio for the purple module (i.e., the x-axis of the plot in Fig. 3e top) whereas it is negative for the blue module (i.e., the y-axis). Second, the eigengene expression differential between the LE and CT groups (symbolized by a Δ_diet_ in Fig. 3e) is similar for both lines confirming the absence of a diet × line interaction. We found the same characteristics for the red vs. turquoise modules (Fig. 3e bottom). This illustrates again that this difference is independent of the line effect, and the absence of interactions at the gene expression level, as already seen in Fig. 1c.

The functional analysis of each co-expressed gene module in the hypothalamus revealed KEGG terms similar to the full list of over- and under-expressed genes for the turquoise and blue modules, respectively, and no KEGG term enrichment for the green, red and yellow modules. In the pink module, three genes were associated with “N-Glycan biosynthesis”, while the brown module was enriched in genes related to vesicles and organelles. Finally, the black module was enriched in terms associated with immunological functions (see Additional file 6). This last module, composed of 134 genes, is associated with 10 immunological-related pathways, supported by 22 genes in total, such as *C1QA*, *C1QB* and *C1QC*, *C3AR1*, *CD14*, *IRF1* and *TLR4*. In the blood, we found seven modules in the list of over-expressed genes and five modules in the list of under-expressed genes. Functional analysis revealed KEGG terms similar to the full list of under-expressed genes for the black module. No KEGG term enrichment were found for the purple, magenta, green, blue, pink, turquoise, brown, and red modules. The greenyellow module was enriched with genes associated to “Ribosome” and “Protein processing in endoplasmic reticulum”, while the salmon module was enriched with 3 genes associated with the “Estrogen signaling pathway” (See Additional file 7).

### Focus on genomic regions concentrating differentially expressed genes

**Fig. 4:**
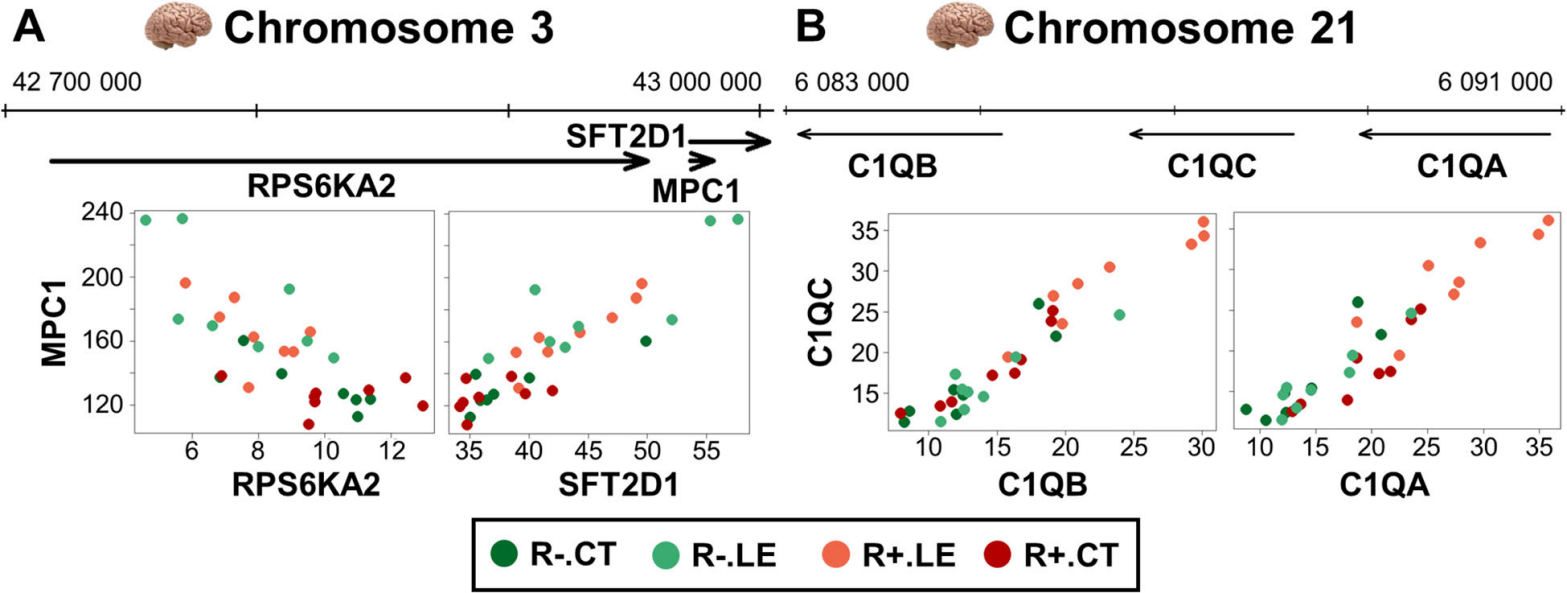
Genomic localization and pairwise scatterplots of expression of four groups of co-expressed and co-localized genes. In each plot, *top:* genomic localization of the three genes. *Bottom*: pairwise scatterplots of expression (FPKM) the genes. **a** cluster composed of *RPS6KA2*, *MPC1* and *SFT2D1*. **b** cluster composed of *C1QA*, *C1QB* and *C1QC*

We searched for groups of three or more DEG in close physical proximity (i.e., side by side) along the genome that had a significant pairwise expression correlation (|*r*| > 0.7 & *p*_*FDR*_ < 10^− 4^), hypothesizing that such genes might be co-regulated by a local common mechanism. We found two such proximal co-expressed gene groups in the hypothalamus (Fig. 4a and b), composed of *RPS6KA2*, *MPC1* and *SFT2D1* for the first one (Fig. 4a) and *C1QA*, *C1QB* and *C1QC* for the second (Fig. 4b), genes that belong to the black WGCNA module, which was enriched in immunity-related genes.

## Discussion

### Layers from both lines adapt to the low-energy diet by increasing feed intake and changing body reserve dynamics

The absence of significant differences in egg production (number and weight) between the LE and CT groups suggests that the animals were able to adapt to a suboptimal diet. The adaptive mechanisms adopted by the animals to compensate for the decrease in diet-energy content involved an increase in feed intake and a decrease of the abdominal adipose tissue. The increase in feed intake in response to a 15%-energy-depleted diet over 14 weeks is consistent with the results from Grobas et al. [1] and Harms et al. [2]. However, this increased ingestion did not allow the layers from the LE group to fully compensate for the difference in energy, (Table 1) as indicated by the significant difference in Energy Intake between the diet groups. The decrease of the percentage of fat weight to the total weight, probably resulting from this incomplete compensation, is consistent with the results reported by Murugesan and Persia [3], where layers were fed a 3%-energy-depleted diet compared to the control over 11 weeks, although the authors did not observe a feed intake modification, perhaps due to the small difference in energy between the two diets.

The absence of a significant line × diet interaction at the expression level is consistent with the absence of interaction at the trait level, meaning that both R+ and R- birds reacted to the energy-depleted diet in a similar way and with the same magnitude. At the expression level, the Δ_diet_ values in Fig. 3e illustrates this conclusion: as an example, Δ_diet_ for the genes belonging to the purple module are similar in the two lines whereas these genes are more expressed in R- than in R+.

### Liver and adipose tissue transcriptomes were unaffected by the low-energy diet

Neither the abdominal adipose tissue nor the liver transcriptomes were affected by the diet change, as shown by the small number of differentially expressed genes in these two tissues (15 and 2, respectively). The absence of differentially expressed genes in the abdominal adipose tissue indicates that the mobilization of body reserves observed with the adipose tissue weight decrease was not mainly driven transcriptionally. This observation is consistent with the fact that the two key genes of adipocytes lipolysis, *PNPLA2* (*alias ATGL*) coding the enzyme catalyzing the initial step of this process and *LIPE* coding the Hormone-Sensitive Lipase which primarily hydrolyzes stored triglycerides to free fatty acids are known to be quickly regulated through post-translational modifications such as phosphorylation [6]. We further confirmed that these two genes were not differentially expressed using RT-qPCR (for *PNPLA2*, ΔCt_LE-CT_ = 0.02, *p* = 0.97 and for *LIPE*, ΔCt_LE-CT_ = 0.21, *p* = 0.50). The mobilized lipids resulting of this probable adipose tissue lipolysis could have been used by the hypothalamus as an energy source, as we discuss later. Concerning the liver, the absence of reaction at the transcriptomic level shows that the difference in energy between the two diets did not impact gene expression, which suggests an absence of hepatic lipid metabolism variation. Indeed, lipid metabolism is known to be highly regulated at the transcriptional level, as previously shown in chickens [5, 7]. In these studies, which explored the impact of the diet fiber and lipid composition variation or the fasting-feeding transition (known to impact hepatic lipid metabolism), numerous genes involved in the lipid metabolism were impacted at the transcriptional level. The result observed here in liver can be explained by the partial compensation of the energy depletion by the increase in feed intake and the mobilization of the body reserves. We confirmed by RT-qPCR the absence of differential expression of *PPARα*, a key genes of fatty acid *β*-oxidation (ΔCt_LE-CT_ = − 0.16, *p* = 0.30) and for *FASN* and *SREBF1*, two key genes of fatty acid synthesis (for *FASN*, ΔCt_LE-CT_ = − 0.24, *p* = 0.37 and for SREBF1, ΔCt_LE-CT_ = − 0.14, *p* = 0.57).

### Blood cells participate in the adaptation to the CT versus LE diet changes

While the liver and adipose tissue were almost unaffected by the low energy diet, at least from the transcriptomic point of view, blood cell genes reacted strongly to the low-energy diet with more than 1000 genes modulated by the diet change but for which the interpretation remains difficult. Indeed, the red blood cell components differ between mammals and vertebrates more distant in the evolutionary scale, such as birds or fish. In these animals, erythrocytes and thrombocytes are nucleated and their transcriptional activity is not yet well defined. Secondly, the blood transcriptome is mainly studied to evaluate the response to an inflammatory and immune challenge and rarely to study the effects of diets. To our knowledge, no study has explored so far the blood transcriptome profile in chicken under such conditions. We found an activation of genes involved in RNA degradation and ribosome activity and a repression of genes involved in cholesterol and amino acid biosynthesis, as well as galactose and fructose metabolisms. Cholesterol synthesis decrease in response to energy restriction was also reported by Bouvier-Muller et al. [8] in energy-restricted ewe’s blood transcriptome. Under-expression of some of the genes described in our study like *CYP51A1*, *DHCR24*, *FDFT1* and *SQLE* was also observed in ewes fed a low energy diet versus control (restriction to 60% of the calculated net energy requirements during 15 days). Furthermore, three genes involved in macrophage cholesterol efflux and transport [9] show a significant, or a trend toward, over-expression in our study: *ABCA1* (FC = 1.68 *p*_*FDR*_ = 0.07) and *APOA1* (FC = 2.10 *p*_*FDR*_ = 0.08), the latter being the chicken equivalent of human *APOE* [10], and *CETP* (FC = 1.61, *p*_*FDR*_ = 0.02). The precise relationship between these genes and their differential expression remains to be linked with the feed intake. Taken together, these reports and our results suggest that the chicken blood transcriptome may play a role in the adaptation of birds to feed stress. However, the differentially expressed genes are quite hard to interpret, and further studies will be required to unveil the mechanisms at play.

### In the hypothalamus, the low-energy diet seems to alter the general synaptic organization, partly through a modulation of cholesterol and a global protein synthesis associated to fatty acid *β*-oxidation

**Fig. 5:**
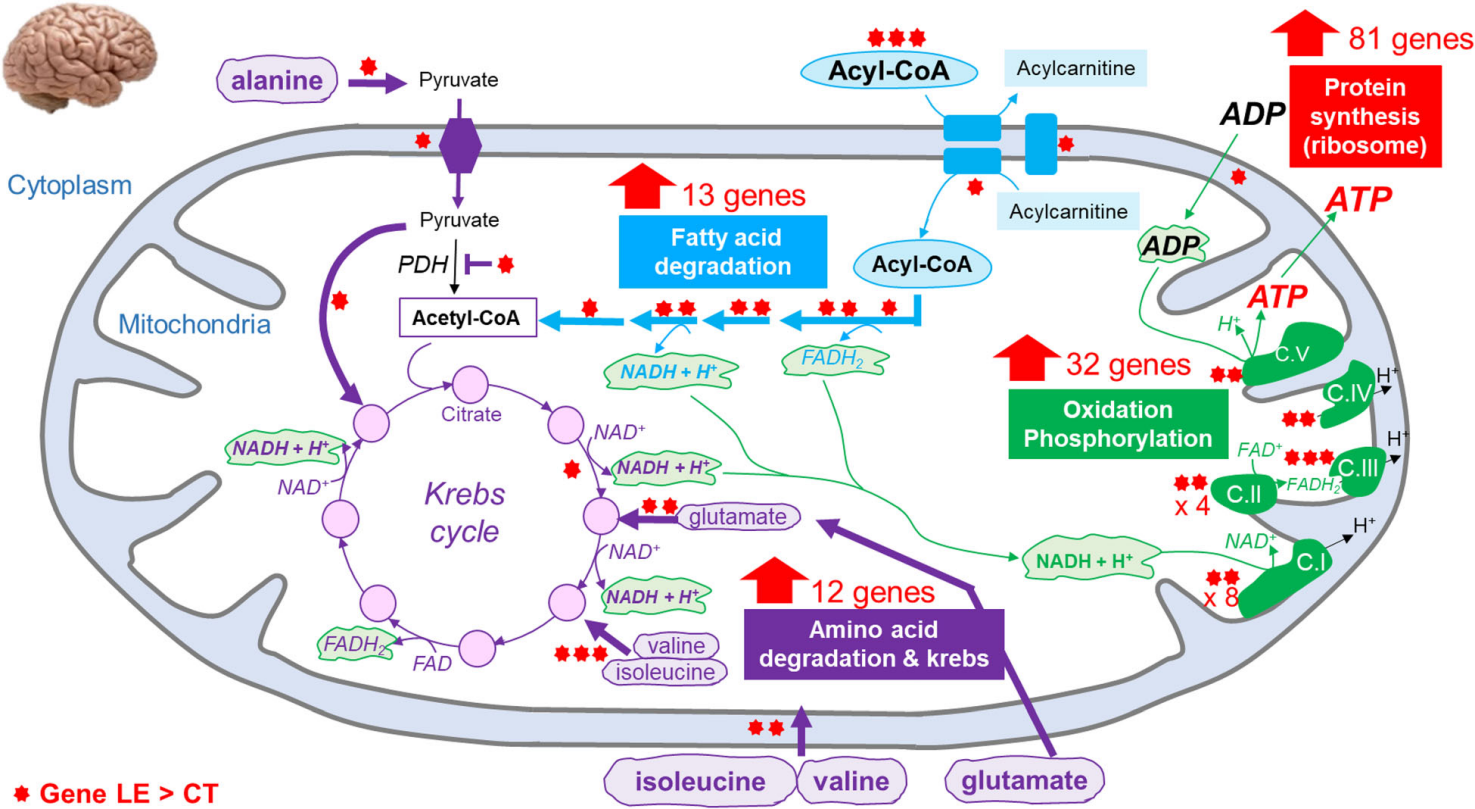
Proposed mechanism of energy pathways increased in the hypothalamic cells in LE diet. In blue: reactions related to fatty acid β-oxidation (*ETFDH*, *ACADL*, *ACADS*, *ECHS1*/*ECI1*, *HADH*, *HADHB*, *ACAA2*), to fatty acids transport through the plasma (*FABP4*, *FABP7*, *SLC27A1*), and the mitochondrial (*CPT2*, *CACT)* membrane. In purple: reactions related to TCA cycle (*IDH2*), to transport of amino-acids (*BCKDHA*, *BCKDHB*) and pyruvate (*MPC1*, *MPC2*) in the mitochondria, to the integration of amino-acids in the TCA cycle as α-ketoglutarate (*GDH1*, *GPT2*) or succinyl-CoA (*ALDH6A1*, *ECHS1*, *HIBDCH*) and of the pyruvate as oxaloacetate (*PC*). In green: reactions related to oxidative phosphorylation and mitochondrial respiratory chain complex I (*MT-ND1*, *MT-ND2*, *MT-ND3*, *MT-ND4*, *ACAD9*, *MT-ND4L*, *MT-ND5*, *MT-ND6*, *NDUFA2*, *NDUFA8*, *NDUFA10*, *NDUFB9*, *NDUFS4*, *NDUFV3*, *FOXRED1*), complex II (*MT-CO1*, *MT-CO2*, *MT-CO3*, *APOPT1*, *COX14*, *COX7B*, *COA5*, *COA6*), complex III (*MT-CYB*, *UQCRB*, *UQCRQ*), complex IV (*MT-ATP6*, *MT-ATP8*) and complex V (*SDHD*, *SDHAF2*), as well as the entry of ADP in the mitochondria (*SLC25A1*)

The hypothalamus is a brain area that integrates metabolic and hormonal cues and controls appetite and peripheral metabolism. It is composed of different cell types, including neurons and “non-neuronal” cells (such as astrocytes, microglial cells, oligodendrocytes and endothelial cells) [11], and the transcriptomic changes observed in this study reflect most likely changes occurring in different cells, but we are unable to distinguish which ones. Notwithstanding, the differential expression analysis suggests an effect of the low-energy diet in neuronal circuits. We detected an under-expression of genes involved in the synaptic vesicle cycle, as well as in the glutamatergic, dopaminergic and GABAergic synapses. In addition, key genes involved in the cholesterol synthesis (*CYP51A1*, *DHCR7*, *DHCR24*, *FDFT1* and *SQLE*) and in the cholesterol efflux from neuronal cells, namely *ABCA7* (FC = 0.67, *p*_*FDR*_ = 0.03) and *ABCG4* (FC = 0.64, *p*_*FDR*_ = 0.007) [12] were also under-expressed. Interestingly, the adult brain is the most cholesterol-rich organ, containing 20% of the whole body’s cholesterol [13]. The majority of it is present in myelin sheaths and the rest in the plasma membranes of astrocytes and neurons to maintain their morphology and synaptic transmission [14]. Taken together, these findings reveal a link between nutrition and brain plasticity in chicken, as it has already been described in mice [15, 16]. Furthermore, our results suggest an overall activation of protein synthesis in the hypothalamic cells, one of the most energy-consuming processes in a cell [17], probably reflecting the protein machinery necessary to promote feed intake increase. Indeed, we detected in the hypothalamus of the low-energy group 83 over-expressed DEG related to the ribosome machinery indicating activation of numerous genes related to the oxidative phosphorylation (that produces ATP) and the fatty acid oxidation (used as fuel for the respiratory chain) (Fig. 5). Concerning the oxidative phosphorylation, we observed 32 over-expressed genes coding the 5 protein complexes located in the inner mitochondrial membrane (Fig. 5) including the ADP/ATP translocase 1 (*SLC25A4*, FC = 1.79, *p*_*FDR*_ = 3.31 × 10^− 06^) required for the entry of ADP (the substrate of the ATPase) in the mitochondria, and considered as a limiting factor of this process. The NADH and FADH_2_ required by the respiratory chain is produced by the mitochondria β-oxidation of fatty acid, which increase is supported by 10 over-expressed DEG (Fig. 5) and by the integration of amino acids in the Krebs cycle as indicated by the 12 over-expressed DEG identified (Fig. 5). While short and medium chain fatty acids appear to enter the brain-blood barrier by simple diffusion through the plasma membrane, long chain fatty acids (> 12 carbons) need transporters to cross the brain-blood barrier. Some of these transporters such as *FABP4, FABP7* [18] and *SLC27A1* [19] were also overexpressed. Cedernaes et al. [20], obtained similar results, although in a different context. The authors observed an over-representation of genes related to oxidative phosphorylation as well as to ribosome sub-units in mice hypothalamus following a fasting period, and others studies [21] made a link between mitochondrial oxidation of fatty acids in the hypothalamus and increase in feed intake.

### Hypothalamic arachidonic acid may be involved in the difference of feed intake between LE and CT groups through mechanisms involving the hypothalamic endocannabinoid and complement systems

**Fig. 6:**
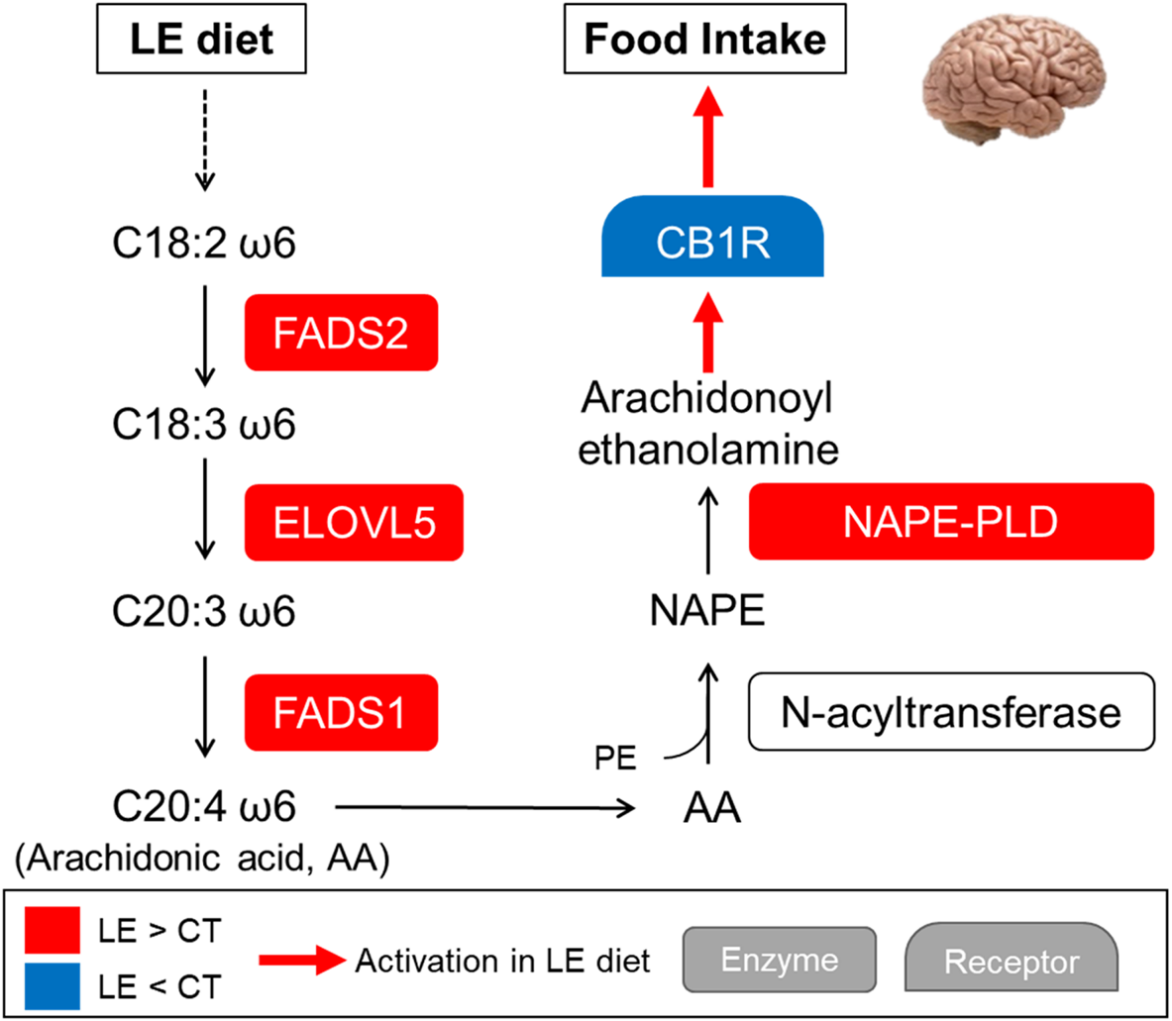
Proposed mechanism leading to an increased feed intake in the LE diet. Diet fatty acids are processed by *FADS1*, *FADS2*, *ELOVL5* and *FADS1*, leading to the production of arachidonic acid (AA). The arachidonic acid eventually lead to the production of Arachidonoyl ethanolamine (AEA), thanks to the action of NAPE-PLD. The AEA acts on CB1R, leading to an increase in feed intake. FADS1 and 2: Fatty Acid Desaturase 1 and 2, ELOVL5: Elongation Of Very Long Chain Fatty Acids Protein 5, NAPE-PLD: N-Acyl Phosphatidylethanolamine Phospholipase D, CB1R: Cannabinoid Receptor 1, AA: Arachidonic Acid, PE: Phosphatidylethanolamine, NAPE: N-arachidonoyl phosphatidylethanolamine, AEA: Arachidonoyl ethanolamine (alias Anandamide)

The involvement of the endocannabinoids in the regulation of feed intake is well documented [22, 23], in particular for the two best known representative of this family of molecules, the 2-AG (2-arachidonoylglycerol) and the Arachidonoyl ethanolamine (AEA also called Anandamine). Both these molecules are the ligands of the endocannabinoid receptor, CN1R. Interestingly, we observed an under-expression of *DAGLB* (FC = 0.74, *p*_*FDR*_ = 0.003), involved in the synthesis of 2-AG [24] and an over-expression of *MGLL* (FC = 1.75, *p*_*FDR*_ = 5.73 × 10^− 06^), coding an enzyme responsible for 2-AG degradation. We also observed an over-expression of *NAPE-PLD* (FC = 1.95, *p*_*FDR*_ = 6.86 × 10^− 11^), which codes for the enzyme that catalyzes the second step of the classical “two-step” pathway of the synthesis of AEA and other NAEs. The first step of this pathway consists in the formation of N-acylphosphatidyl ethanolamines (NAPEs) by the transfer of the acyl chain of phospholipids on phosphatidylethanolamine by a calcium-dependant transacylase [25]. *NAPE-PLD* then catalyses the cleavage of NAPEs to yield NAEs. Different NAEs are generated depending on the nature of the acyl chain in the first step. For example, Arachidonoyl ethanolamine (AEA) derives from the ω6 poly-unsaturated fatty acid (PUFA) arachidonate and Palmitoyl ethanolamide (PEA) derives from the saturated fatty acid palmitate [26]. We observed an over-expression of *FADS1* (FC = 1.99, *p*_*FDR*_ = 3.25 × 10^− 14^), *FADS2* (FC = 2.07, *p*_*FDR*_ = 3.15 × 10^− 10^), *ELOVL2* (FC = 1.87, *p*_*FDR*_ = 0.003) and *ELOVL5* (FC = 1.48, *p*_*FDR*_ = 0.0004), key genes of the PUFA ω6 synthesis [27]. FADS2 catalyzes the Δ6-desaturation of the essential fatty acid linoleic acid (C18:2 ω6) into γ-linolenic acid (C18:3ω6), which is elongated into C20:3 ω6 by ELOVL5; the C20:3 ω6 is then Δ5-desaturated into arachidonic acid (C20:4 ω6) by FADS1 [28] which may lead to the formation of AEA [29]. As 2-AG, AEA could also activates the CB1R endocannabinoid receptor, leading to an increase of feed intake [30]. Consistently with this hypothesis, we observed an under-expression of CB1R which might be due to a negative feedback following CB1R activation. Figure 6 summarizes this proposed mechanism. Interestingly, we found that *FADS2* and *NAPE-PLD* were highly correlated to *NR1H3 (*alias *LXRα*) that codes for a receptor involved in the control of various physiological functions with a major role in fatty acid homeostasis and cholesterol metabolism [31]. The mechanism of the regulation of the *FADS2* and *NAPE-PLD* transcription that can be direct or indirect, remains to be elucidated. Interestingly, the arachidonic acid is also a precursor of the prostaglandins [32], which has been shown to be involved in feed intake regulation along with complement system molecules [33].

Among the eight modules detected by WGCNA using the lists of hypothalamic DEG, the black module was composed of over-expressed genes related to immunity. Three of them, *C1QA*, *C1QB* and *C1QC* were detected as co-localized and co-expressed genes. The co-localization and strong co-expression of *C1QA*, *C1QB* and *C1QC* strongly suggest a mechanism of common regulation. These three genes code for the A, B and C polypeptide chains composing the C1q molecule, a subcomponent of the C1 complex involved in the complement activation [34]. The complement system is a part of the innate immune system, involved in the host defense against bacteria and in the removal of wastes [35]. C3AR1, the receptor of C3a, which is produced upon the activation of the complement system [36], also belongs to the black co-expression module. Interestingly, Ohinata et al. showed that an agonist of C3AR could suppress feed intake in mice [37] through prostaglandin (PG) E_2_ production [33]. Furthermore, the same authors showed that C5a, another member of the complement system, stimulated feed intake via a mechanism involving this time PGD_2_ [38]. Interestingly, as we discussed earlier, we found that key genes of the poly-unsaturated fatty acid (PUFA) ω6 synthesis, that lead to the formation of arachidonic acid, the precursor of prostaglandins, were overexpressed in LE group. Finally, *C1QTNF4* (C1q/TNF-related Protein 4), that possesses two tandem globular C1q domains and is under-expressed in LE versus CT (FC = 0.65, *p*_*FDR*_ = 0.01), has also been shown to suppress feed intake in mice [39]. Surprisingly, we found only one other group of 3 co-localized and co-expressed genes in the hypothalamus DEG lists. Such results show that regulatory mechanisms affecting different genes located in a same genomic region are not so frequent in response to a diet change despite the high number of DEG identified and analyzed in the hypothalamus and the blood. We found similar results in a previous study that evaluated on the impact of diet-composition change on the breast muscle, adipose tissue and liver of broiler, in which one region was identified [5].

## Conclusions

This work is the first to provide a multi-tissue analysis of layers submitted to a hypo-energetic diet on a long period. Neither the adipose tissue nor the liver seemed to be affected by the diet change at the transcriptional level, suggesting regulations occurring at a different level. In contrast, we observed a strong effect of the diet on the hypothalamic transcriptome of the layers. The regulation of feed intake in the hypothalamus is a complex mechanism. Our results in chicken suggest, as in mice, a link between feed intake and brain plasticity, as well as fatty acid metabolism [40–43]. We show here a mechanism in chickens that seems to modify feeding behavior through an increase in feed intake in response to a low-energy diet, allowing egg mass production to be maintained, probably through the action of the endocannabinoid and the complement systems that involve the hypothalamic poly-unsaturated fatty acid synthesis, and in particular the arachidonic acid. Overall, this work contributes to a better understanding of the adaptive strategies employed by chickens to cope with a suboptimal diet and the impact that this suboptimal feeding may have on egg quality and production. Such understanding is of importance in the frame of the globalized poultry market, in which commercial animals are exposed to a wide diversity of production conditions.

## Methods

### Animals and diet

Laying hens were hatched at the INRA Pôle d’Expérimentation Avicole de Tours (PEAT) in Nouzilly, France. They belonged to two Rhode Island Red layer lines that underwent a 40-year diverging selection on residual feed intake (RFI) [4]. The RFI represents the difference between the observed and the predicted feed consumption based on a multiple regression equation taking into account the average body weight, the weight variation and, for females, the mass of eggs produced over a given period [44, 45]. The R+ chickens were selected to have a positive RFI, reflecting a low feed efficiency, while the R- chickens were selected to have a negative RFI and therefore to be feed efficient. They were reared under standard farming conditions in floor pens until 17 weeks of age. At this age, 45 R+ and 51 R- hens were transferred in individual cages and reared under thermo-neutral conditions (22 °C), with a lighting regimen set at 14 h of light per day and an ad libitum feeding. Of these, 34 R+ and 36 R- hens were fed a commercial diet (control group, CT) and 11 R+ and 15 R- were fed a low-energy diet (low-energy group, LE). The two diets had a similar protein content, while the energy content was reduced by 15% in the LE diet as compared to the standard diet (2450 kcal/kg versus 2880 kcal/kg), due to the replacement of soybean and maize by rapeseed and raw wheat, and by increasing the raw cellulose percentage (7.4 g/kg against 2.6 g/kg). The composition of both diets is detailed in Additional file 8.

### Tissue sampling

At 31 weeks, eight animals from each line (R- and R+) and from each diet (CT and LE) were selected as representative of the group for slaughtering, that is 8 × 2 × 2 = 32 animals. Layers were slaughtered at the fed status by neck cut and bleeding, immediately after head electrical stunning. Right after slaughter, abdominal adipose tissue, the extremity of the left liver lobe and hypothalamus were sampled, snap frozen in liquid nitrogen and stored at − 80 °C until analysis. Blood samples from the same animals were collected from the occipital sinus in EDTA tubes and 100 μL of blood were removed and diluted in 1 mL of TRIzol® reagent (Invitrogen, California, USA). After a vigorous agitation, the tube was maintained at room temperature for five minutes, then quickly frozen in liquid nitrogen and stored at − 80 °C until RNA extraction.

### Traits collection and analysis

Seven traits related to performance and body composition were recorded for the 45 R+ (34 CT and 11 LE) and 51 R- (36 CT and 15 LE) birds. Egg number was recorded from the date of the first egg (around 21 weeks of age) to 31 weeks of age and laying rate (i.e. number of egg laid during the recording period divided by the length of the period in day, expressed in %) was calculated; egg weight (g), static stiffness (N.mm^− 1^) were calculated from 3 eggs per hen collected at 30 weeks of age, and abdominal adipose was weighted at slaughter. Weekly feed intake was measured over 4 weeks, from 27 to 31 weeks of age and body weight (g) at 31 weeks of age. Residual feed intake was computed as described in Bordas et al. [4]. Traits were analyzed with R version 3.4.2 [46]. A two-way analysis of variance was performed with line, diet and the interaction between line and diet as main effects using the R function lm, and the R package “car” [47].

### RNA isolation

Approximately 100 mg of adipose tissue and 30 mg of liver were homogenized in TRIzol® reagent (Invitrogen, California, USA), and the whole blood mixed with 1 mL of TRIzol® was adjusted between 4 and 4.5 with 10 μL of 5 N glacial acetic acid [48]. The total RNA was then extracted according to the manufacturer’s instructions, resuspended in 50 μL of RNA-free water and stored at − 80 °C. For the hypothalamus, we used the kit Allprep DNA/RNA (Qiagen). The RNA was extracted from the hypothalamus according to the manufacturer’s instructions. The total RNA was quantified with a NanoDrop® ND-1000 spectrophotometer (Thermo Scientific, Illkirch, France). The RNA quality was controlled using an Agilent 2100 bioanalyzer (Agilent Technologies France, Massy, France). The average RNA integrity numbers were 7.3 ± 0.6 (mean ± SD) for the adipose tissue, 8.8 ± 0.48 for the hypothalamus, 8.2 ± 0.5 for the whole blood and 9.2 ± 0.3 for the liver.

### RNA-seq data acquisition

Paired-end sequencing was conducted on all samples using an Illumina HiSeq3000 (Illumina, California, USA) system, with 2 × 150 bp. Libraries with an on average 465-bp insert were prepared following Illumina’s instructions by purifying poly-A RNAs (TruSeq RNA Sample Prep Kit). Illumina adapters containing indexing tags were added for subsequent identification of samples. Samples were PCR-amplified, and quantitative PCR was then performed for library quantification (QPCR NGS Library Quantification kit). Eight samples were filled on one lane within a flow cell with 2 samples for each of the four line × diet groups to minimize the inter-lane bias. After sequencing, the indexed adapter sequences were trimmed using CASAVA v.1.8.2 software (Illumina). We obtained an average of 90 million reads per sample (84 million for the adipose tissue, 98 million for the blood, 86 million for the hypothalamus and 90 million for the liver), for a grand total of 11 billion reads. For each sample, reads were mapped to the *Gallus gallus*-5 reference genome using STAR v.2.3.0e [49]. PCR duplicates were removed using rmdup tool from SAMtools suite [50]. For each sample, quantification was performed using RSEM [51] with the Ensembl v93 annotation.

### RNA-seq data analysis

All the analyses were performed with R version 3.4.2. The trimmed mean of M-values (TMM) scaling factor method was used for library size normalization [52] using the R/Bioconductor package edgeR [53] version 3.12.1. In each tissue, the expressed genes were selected if their FPKM expressions were over 0.1 in at least 80% of the samples of a group line × diet (FPKM expression being obtained after TMM normalization using “rpkm” function from edgeR package). Differential expression analysis was performed using the R/Bioconductor package edgeR [53] based on a generalized negative binomial model for model fitting. We used the “edgeR-Robust” method to account for potential outliers when estimating per gene dispersion parameters [54]. *P*-values were corrected for multiple testing using the Benjamini-Hochberg approach [55] to control the false discovery rate (FDR), and genes were identified as significantly differentially expressed if *p*_*FDR*_ < 0.05.

### Functional enrichment analysis

The enrichment analysis of Kyoto Encyclopedia of Genes and Genomes (KEGG) terms in each list of interest of differentially expressed genes was performed using the STRING tool [56] (https://string-db.org). Only the 1-to-1 human orthologous genes with a standardized HGNC name were submitted for the analysis, i.e. 67.4% of the 18,346 protein-coding genes of chicken Ensembl v93 annotation.

### Co-expression module detection with WGCNA

We used the R package WGCNA [57] to detect co-expression modules based on gene expression data and a weighted correlation network. Briefly, WGNCA screens for clusters (called modules) of highly correlated genes in the expression dataset. Indeed, while within a list of over- or under-expression in one condition versus another one, one can expect all the genes to be positively correlated to one another, such list can be split into modules of genes with a higher expression correlation among them than with the rest of the list. These genes are more likely to share a common regulation and a common biological function and therefore may highlight more specifically one pathway. In addition, it may happen that a gene subset is not correlated with the other subsets of the same DEG list because of factors other than the one used for the differential expression analysis. These modules are summarized by an eigengene, which corresponds to the first principal component of the module. These eigengenes enable comparisons between modules, clustering of modules, and calculations of correlations between modules and phenotypes. Modules hierarchical clustering was realized using as “1 – the pearson correlation” between modules as distance criterion and “ward’s” method as aggregation criterion.

### Detection of co-localized differentially expressed genes

We used R home-made script to screen for groups of three or more differentially expressed genes, located side-by-side, without consideration for distance, and with a significant pairwise Spearman expression correlation (|*r*| > 0.7 and *p*_*FDR*_ < 10^− 4^).

### RT-qPCR analysis

Reverse transcription (RT) was carried out using the high- capacity cDNA archive kit (Applied Biosystems, Foster City, CA) according to the manufacturer’s protocol. Briefly, reaction mixture containing 2 μL of 10× RT buffer, 0,8 μL of 25X dNTPs, 2 μL of 10X random primers, 1 μL of MultiScribe Reverse Transcriptase (50 U/ μL), and total RNA (2 μg) was incubated for 10 min at 25 °C followed by 2 h at 37 °C and 5 min at 85 °C. Dilution RT reaction was further used for real time quantitative PCR (qPCR). 5 μl of cDNA samples were mixed with 7,5 μl of Sso Advanced Universal SYBR Green Supermix (Bio-Rad), 1,5 μl H20 and 330 nM of specific reverse and forward primers. Reaction mixtures were incubated in an CFX connect Real-Time PCR Detection System (Bio-Rad, Marne la Coquette, France) programmed to conduct one cycle (95 °C for 30 s) and 43 cycles (95 °C for 15 s and 60 °C for 30 s). A melting curve program was then performed for each gene to check the presence of a unique product with specific melting temperature. For each sample and each gene, PCR runs were performed in duplicates. The sequences of the primers used were, from 5′ to 3′: *LIPE*, forward “GTCTCGGGTTCCAGTTCGTG”, reverse “CGTAGGACACCAACCCGATG”. *PNPLA2*, forward “TGGGCAGTCATCTTTCAGCCA”, reverse “AAGCTGACGCTGGTACTCCT”. *FASN*, forward “TGAAGGACCTTATCGCATTGC”, reverse “GCATGGGAAGCATTTTGTTGT”. *PPARα*, forward “GTCGCTGCCATCATTTGCTGT”, reverse “TTGCCGGAGGTCAGCCATTT”. SREBF1, forward “GTCGGCGATCCTGAGGAA”, reverse “CTCTTCTGCACGGCCATCTT”.

## Supplementary information

**Additional file 1.** Means (±SD) and significance for Body weight at 17 weeks for the effect of the diet, the line and their interaction.

**Additional file 2.** List of the genes uniquely expressed in each tissue.

**Additional file 3.** List of the differentially expressed genes (LE vs. CT) in each tissue.

**Additional file 4.** List of the 26 and 44 KEGG pathways significantly enriched in the over- and under-expressed genes in the hypothalamus.

**Additional file 5.** List of the 2 and 8 KEGG pathways significantly enriched in the over- and under-expressed genes in the blood.

**Additional file 6.** List of the KEGG pathways significantly associated with WGCNA’s modules detected using hypothalamic DEG.

**Additional file 7.** List of the KEGG pathways significantly associated with WGCNA’s modules detected using blood DEG.

**Additional file 8.** Composition of the diets.

## Data Availability

The 64 RNA-seq samples are available in European Nucleotide Archive (ENA) through ENA Series accession number PRJEB28745.

## Abbreviations

AA: Arachidonic Acid
ACAA1: Acetyl-CoA Acyltransferase 1
ACADL: Acyl-CoA Dehydrogenase Long Chain
ACADS: Acyl-CoA Dehydrogenase Short Chain
ACO2: Aconitase 2
ACOX2: Acyl-CoA Oxidase 2
ACSBG1: Acyl-CoA Synthetase Bubblegum Family Member 1
AEA: Arachidonoyl ethanolamine (alias Anandamide)
ALDH7A1: Aldehyde Dehydrogenase 7 Family Member A1
APOA1 and APOC3: Apolipoproteins
ATP5G1: ATP Synthase Membrane Subunit C Locus 1
ATP5H: ATP Synthase Peripheral Stalk Subunit D
ATP5J: ATP Synthase Peripheral Stalk Subunit F6
C1QA, C1QB and C1QC: Complement C1q chains
C3AR1: Complement C3a Receptor 1
CB1R: Cannabinoid Receptor 1
CD14: CD14 Molecule
CPS1: Carbamoyl-Phosphate Synthase 1
CPT2: Carnitine Palmitoyltransferase 2
CT: Control
CTH: Cystathionine Gamma-Lyase
CYP51A1: Cytochrome P450 Family 51 Subfamily A Member 1
DBI (alias ACBP): Diazepam Binding Inhibitor (alias Acyl-CoA Binding Protein)
DEG: Differentially Expressed Genes
DHCR24: 24-Dehydrocholesterol Reductase
EDC3: Enhancer Of MRNA Decapping 3
ELOVL2 and ELOVL5: Elongation Of Very Long Chain Fatty Acids Protein
ENO2: Enolase 2
EXOSC5: Exosome Component 5
FABP7: Fatty Acid Binding Protein 7
FADS1 and FADS2: Fatty Acid Desaturases
FDFT1: Farnesyl-Diphosphate Farnesyltransferase 1
FDR: False Discovery Rate
FPKM: Fragment Per Kilobase Million
GABA: gamma-Aminobutyric acid
GOT1: Glutamic-Oxaloacetic Transaminase 1
HIF1: hypoxia-inducible factor-1
IRF1: Interferon Regulatory Factor 1
KEGG: Kyoto Encyclopedia of Genes and Genomes
LE: Low-Energy
ME1: Malic Enzyme 1
MRPL: Mitochondrial Ribosomal Protein L
MRPS: Mitochondrial Ribosomal Protein S
MT-CO1 to MT-CO3: Mitochondrially Encoded Cytochrome C Oxidases
MT-CYB: Mitochondrially Encoded Cytochrome B
MT-ND1 to MT-ND6: Mitochondrially Encoded NADH:Ubiquinone Oxidoreductase Core Subunits
NAE: N-acyl ethanolamine
NAPE: N-arachidonoyl phosphatidylethanolamine
NAPE-PLD: N-Acyl Phosphatidylethanolamine Phospholipase D
NR1H3 (alias LXRα): Nuclear Receptor Subfamily 1 Group H Member 3 (alias Liver X Nuclear Receptor alpha)
NSDHL: NAD(P) Dependent Steroid Dehydrogenase-Like
PABPC1: Poly(A) Binding Protein Cytoplasmic 1
PAN2 and PAN3: Poly(A) Specific Ribonuclease Subunit PANs
PE: Phosphatidylethanolamine
PEA: Palmitoylethanolamide
PFKP: Phosphofructokinase, Platelet
PLPP5: Phospholipid Phosphatase 5
RFI: Residual Feed Intake
RPL: Ribosomal Protein L
RPS: Ribosomal Protein S
RQCD1: CCR4-NOT Transcription Complex Subunit 9
SCP2: Sterol Carrier Protein 2
SDHD: Succinate Dehydrogenase Complex Subunit D
SKIV2L: Ski2 Like RNA Helicase
SLC27A1: Solute Carrier Family 27 Member 1
SQLE: Squalene Epoxidase
TALDO1: Transaldolase 1
TKT: Transketolase
TLR4: Toll Like Receptor 4
TMM: Trimmed Mean of M-values
TOB2: Transducer Of ERBB2, 2
TPI1: Triosephosphate Isomerase 1
WGCNA: Weighted Gene Co-expression Network Analysis
WHSC1L1 (alias NSD3): Nuclear Receptor Binding SET Domain Protein 3

## References

[1] Grobas S, Mendez J, De Blas C, Mateos G (1999). Laying hen productivity as affected by energy, supplemental fat, and linoleic acid concentration of the diet. Poult Sci.

[2] Harms RH, Russell GB, Sloan DR (2000). Performance of four strains of commercial layers with major changes in dietary energy. J Appl Poult Res.

[3] Murugesan GR, Persia ME (2013). Validation of the effects of small differences in dietary metabolizable energy and feed restriction in first-cycle laying hens. Poult Sci.

[4] Bordas A, Tixier-Boichard M, Merat P (1992). Direct and correlated responses to divergent selection for residual food intake in Rhode island red laying hens. Br Poult Sci.

[5] Desert C, Baéza E, Aite M, Boutin M, Le Cam A, Montfort J, et al. Multi-tissue transcriptomic study reveals the main role of liver in the chicken adaptive response to a switch in dietary energy source through the transcriptional regulation of lipogenesis. BMC Genomics. 2018;19. 10.1186/s12864-018-4520-5. 10.1186/s12864-018-4520-5 PMC5842524 29514634

[6] Kim S-J, Tang T, Abbott M, Viscarra JA, Wang Y, Sul HS (2016). AMPK phosphorylates Desnutrin/ATGL and hormone-sensitive lipase to regulate lipolysis and fatty acid oxidation within adipose tissue. Mol Cell Biol.

[7] Désert C, Duclos MJ, Blavy P, Lecerf F, Moreews F, Klopp C (2008). Transcriptome profiling of the feeding-to-fasting transition in chicken liver. BMC Genomics.

[8] Bouvier-Muller J, Allain C, Tabouret G, Enjalbert F, Portes D, Noirot C, et al. Whole blood transcriptome analysis reveals potential competition in metabolic pathways between negative energy balance and response to inflammatory challenge. Sci Rep. 2017;7. 10.1038/s41598-017-02391-y. 10.1038/s41598-017-02391-y PMC5443788 28539586

[9] Tall AR, Costet P, Wang N (2002). Regulation and mechanisms of macrophage cholesterol efflux. J Clin Invest.

[10] Rajavashisth TB, Dawson PA, William DL, Shackelford JE, Lebherz H, Lusis AJ. Structure, evolution, and regulation of chicken apolipoprotein A-I. J Biol Chem. 1987;262:7058–7065. 3108248

[11] Freire-Regatillo A, Argente-Arizón P, Argente J, García-Segura LM, Chowen JA. Non-neuronal cells in the hypothalamic adaptation to metabolic signals. Front Endocrinol. 2017;8. 10.3389/fendo.2017.00051. 10.3389/fendo.2017.00051 PMC5359311 28377744

[12] Kim WS, Weickert CS, Garner B (2008). Role of ATP-binding cassette transporters in brain lipid transport and neurological disease. J Neurochem.

[13] Björkhem I, Meaney S (2004). Brain cholesterol: long secret life behind a barrier. Arterioscler Thromb Vasc Biol.

[14] Dietschy JM, Turley SD (2004). Thematic review series: brain lipids. Cholesterol metabolism in the central nervous system during early development and in the mature animal. J Lipid Res.

[15] Pinto S, Roseberry AG, Hongyan L, Diano S, Shanabrough M, Cai X (2004). Rapid rewiring of Arcuate nucleus feeding circuits by Leptin. Science.

[16] Nuzzaci D, Laderrière A, Lemoine A, Nédélec E, Pénicaud L, Rigault C, et al. Plasticity of the Melanocortin system: determinants and possible consequences on food intake. Front Endocrinol. 2015;6. 10.3389/fendo.2015.00143. 10.3389/fendo.2015.00143 PMC4568417 26441833

[17] Buttgereit F, Brand MD (1995). A hierarchy of ATP-consuming processes in mammalian cells. Biochem J.

[18] Mitchell RW, Hatch GM (2011). Fatty acid transport into the brain: of fatty acid fables and lipid tails. Prostaglandins. Prostaglandins Leukot Essent Fat Acids.

[19] Mitchell RW, On NH, Del Bigio MR, Miller DW, Hatch GM (2011). Fatty acid transport protein expression in human brain and potential role in fatty acid transport across human brain microvessel endothelial cells: fatty acid transport protein expression in human brain. J Neurochem.

[20] Cedernaes Jonathan, Huang Wenyu, Ramsey Kathryn Moynihan, Waldeck Nathan, Cheng Lei, Marcheva Biliana, Omura Chiaki, Kobayashi Yumiko, Peek Clara Bien, Levine Daniel C., Dhir Ravindra, Awatramani Raj, Bradfield Christopher A., Wang Xiaozhong A., Takahashi Joseph S., Mokadem Mohamad, Ahima Rexford S., Bass Joseph (2019). Transcriptional Basis for Rhythmic Control of Hunger and Metabolism within the AgRP Neuron. Cell Metabolism.

[21] Dietrich MO, Horvath TL (2013). Hypothalamic control of energy balance: insights into the role of synaptic plasticity. Trends Neurosci.

[22] Di Marzo V, Matias I (2005). Endocannabinoid control of food intake and energy balance. Nat Neurosci.

[23] Bermudez-Silva FJ, Viveros MP, McPartland JM (2010). Rodriguez de Fonseca F. the endocannabinoid system, eating behavior and energy homeostasis: the end or a new beginning?. Pharmacol Biochem Behav.

[24] Murataeva N, Straiker A, Mackie K (2014). Parsing the players: 2-arachidonoylglycerol synthesis and degradation in the CNS: 2-AG synthesis and degradation in the CNS. Br J Pharmacol.

[25] Ezzili C, Otrubova K, Boger DL (2010). Fatty acid amide signaling molecules. Bioorg Med Chem Lett.

[26] Bowen KJ, Kris-Etherton PM, Shearer GC, West SG, Reddivari L, Jones PJH (2017). Oleic acid-derived oleoylethanolamide: a nutritional science perspective. Prog Lipid Res.

[27] Guillou H, Zadravec D, Martin PGP, Jacobsson A (2010). The key roles of elongases and desaturases in mammalian fatty acid metabolism: insights from transgenic mice. Prog Lipid Res.

[28] de Antueno RJ, Knickle LC, Smith H, Elliot ML, Allen SJ, Nwaka S (2001). Activity of human Δ5 and Δ6 desaturases on multiple n-3 and n-6 polyunsaturated fatty acids. FEBS Lett.

[29] Devane WA, Axelrod J (1994). Enzymatic synthesis of anandamide, an endogenous ligand for the cannabinoid receptor, by brain membranes. Proc Natl Acad Sci.

[30] Jamshidi N, Taylor DA (2001). Anandamide administration into the ventromedial hypothalamus stimulates appetite in rats. Br J Pharmacol.

[31] Ducheix S, Montagner A, Theodorou V, Ferrier L, Guillou H (2013). The liver X receptor: a master regulator of the gut–liver axis and a target for non alcoholic fatty liver disease. Biochem Pharmacol.

[32] Harizi H, Corcuff J-B, Gualde N (2008). Arachidonic-acid-derived eicosanoids: roles in biology and immunopathology. Trends Mol Med.

[33] Ohinata K, Yoshikawa M (2008). Central prostaglandins in food intake regulation. Nutrition.

[34] Kishore U, Reid KBM (2000). C1q: structure, function, and receptors. Immunopharmacology..

[35] Noris M, Remuzzi G (2013). Overview of complement activation and regulation. Semin Nephrol.

[36] Sjöberg AP, Trouw LA, Blom AM (2009). Complement activation and inhibition: a delicate balance. Trends Immunol.

[37] Ohinata K, Suetsugu K, Fujiwara Y, Yoshikawa M (2007). Suppression of food intake by a complement C3a agonist [Trp5]-oryzatensin (5–9). Peptides.

[38] Ohinata K, Takagi K, Biyajima K, Kaneko K, Miyamoto C, Asakawa A (2009). Complement C5a stimulates food intake via a prostaglandin D2- and neuropeptide Y-dependent mechanism in mice. Prostaglandins Other Lipid Mediat.

[39] Byerly MS, Petersen PS, Ramamurthy S, Seldin MM, Lei X, Provost E (2014). C1q/TNF-related protein 4 (CTRP4) is a unique secreted protein with two tandem C1q domains that functions in the hypothalamus to modulate food intake and body weight. J Biol Chem.

[40] Loftus TM (2000). Reduced food intake and body weight in mice treated with fatty acid synthase inhibitors. Science..

[41] Obici S, Feng Z, Arduini A, Conti R, Rossetti L (2003). Inhibition of hypothalamic carnitine palmitoyltransferase-1 decreases food intake and glucose production. Nat Med.

[42] Minokoshi Y, Alquier T, Furukawa N, Kim Y-B, Lee A, Xue B (2004). AMP-kinase regulates food intake by responding to hormonal and nutrient signals in the hypothalamus. Nature..

[43] López M, Varela L, Vázquez MJ, Rodríguez-Cuenca S, González CR, Velagapudi VR (2010). Hypothalamic AMPK and fatty acid metabolism mediate thyroid regulation of energy balance. Nat Med.

[44] Byerly TC, Kessler JW, Gous RM, Thomas OP (1980). Feed requirements for egg production. Poult Sci.

[45] Bordas A, Merat P (1981). Genetic variation and phenotypic correlations of food consumption of laying hens corrected for body weight and production. Br Poult Sci.

[46] R Core Team (2017). R: A language and environment for statistical computing.

[47] Fox J, Weisberg S (2011). An R companion to applied regression. Second.

[48] Chiari Y, Galtier N (2011). RNA extraction from sauropsids blood: evaluation and improvement of methods. Amphibia-Reptilia.

[49] Dobin A, Davis CA, Schlesinger F, Drenkow J, Zaleski C, Jha S (2013). STAR: ultrafast universal RNA-seq aligner. Bioinformatics.

[50] Li H, Handsaker B, Wysoker A, Fennell T, Ruan J, Homer N (2009). The sequence alignment/map format and SAMtools. Bioinformatics..

[51] Li B, Dewey CN (2011). RSEM: accurate transcript quantification from RNA-Seq data with or without a reference genome. BMC bioinformatics.

[52] Robinson MD, Oshlack A (2010). A scaling normalization method for differential expression analysis of RNA-seq data. Genome Biol.

[53] Robinson MD, McCarthy DJ, Smyth GK (2010). edgeR: a bioconductor package for differential expression analysis of digital gene expression data. Bioinformatics..

[54] Zhou X, Lindsay H, Robinson MD (2014). Robustly detecting differential expression in RNA sequencing data using observation weights. Nucleic Acids Res.

[55] Benjamini Y, Hochberg Y (1995). Controlling the false discovery rate: a practical and powerful approach to multiple testing. J R Stat Soc Ser B Methodol.

[56] Szklarczyk D, Franceschini A, Wyder S, Forslund K, Heller D, Huerta-Cepas J (2015). STRING v10: protein–protein interaction networks, integrated over the tree of life. Nucleic Acids Res.

[57] Langfelder P, Horvath S (2008). WGCNA: an R package for weighted correlation network analysis. BMC Bioinformatics.

